# Empathy level towards patients among thai dental students: a cross-sectional study

**DOI:** 10.1186/s12903-023-02891-6

**Published:** 2023-03-30

**Authors:** Palinee Detsomboonrat, Sudthida Theppanich, Suttida Banyen, Sansern Hongviphat, Yutthana Khamnil, Komsun Lapauthaya, Anupap Somboonsavatdee, Saw Nay Min

**Affiliations:** 1 Department of Community Dentistry, Faculty of Dentistry, Chulalongkorn University, Bangkok, Thailand; 2 Undergraduate dental student, Faculty of Dentistry, Chulalongkorn University, Bangkok, Thailand; 3 Phrasamutjedee Hospital, Samutprakan province, Thailand; 4 Department of Community Dentistry, Faculty of Dentistry, Mahidol University, Bangkok, Thailand; 5 Department of Statistics, Chulalongkorn Business School, Chulalongkorn University, Bangkok, Thailand

**Keywords:** Dental students, Empathy, Jefferson Scale of Empathy, Reliability

## Abstract

**Background:**

This study aimed to develop the Jefferson scale of Empathy - Health Professions student version (JSE-HPS) for the dental student in the Thai version and assess the empathy level in students across gender, universities, and year of dental education.

**Methods:**

JSE-HPS original version was translated to develop the draft Thai JSE-HPS version and was administered to 5 dental students for a pilot test. The final questionnaires (JSE-HPS) were completed by 439 dental students from five public universities and one private in Thailand in the 2021–2022 academic year. The internal consistency and reliability (test-retest) of the questionnaires were tested by using Cronbach’s alpha and intraclass correlation coefficient (ICC). Factor analysis was used to examine the underlying factors of the JSE-HPS (Thai language).

**Results:**

The JSE-HPS represented good internal consistency (Cronbach’s α = 0.83). Factor analysis revealed, “Compassionate Care”, “Perspective Taking” and “Ability to stand in Patients’ Shoes” as the first, second, and third factors, respectively. The mean empathy score of dental students was 114.30 (SD = 13.06) from the total score of 140. There were no significant differences in the empathy levels among genders, study programs, grades, universities, regions, types of universities, and years of study.

**Conclusion:**

The findings confirm the reliability and validity of the JSE-HPS (Thai version) to measure the empathy level among dental students. Integrating empathic elements into the dental curriculum will help student learning to be more effective and improve treatment outcomes.

**Supplementary Information:**

The online version contains supplementary material available at 10.1186/s12903-023-02891-6.

## Background

An emerging paradigm views empathy as one of the important attributions of medical professionals and is related to health care quality in clinical practice [1]. Empathy, a part of the physician-patient relationship, affects positively the outcomes of psychosocial related factors such as fear, anxiety, life quality, and on assessable outcome indicators which are the symptom, alleviation of pain, and lessening recovery time [2, 3]. Empathy means “feeling into” which was adapted from “em” and “pathos” in Greek terms [4]. Empathy is described as a set of cognitive characteristics that comprise the ability to identify and understand the circumstances, pain, suffering, and perceptions of patients, as well as the ability to express an understanding of these feelings and perceptives back to them [2].

Evidence-based studies have shown that greater empathy can produce better results in treatment outcomes as well as higher patient satisfaction rates, and reduce the chance of medical litigation [5]. Similarly, dentists with higher empathy can build more mutual trust, decrease dental fear [6], provide clear and sufficient information for each patient, and improve treatment success [7]. The American Dental Education Association listed empathy as crucial clinical expertise for the education of dental students due to its important role in the dental setting [3].

Thus, the evaluation of empathy levels among the students and providing the appropriate education program in the curriculum have been increasingly focused on in medical and dental schools [8]. However, it still remains a challenge to develop an appropriate measurement tool for assessing the empathy degree in medical students [3]. Even though twenty measures have been applied to evaluate the empathy stage of healthcare professionals, the Jefferson scale of Physician Empathy has been particularly set up in the framework of patient care and the doctor-patient relationship [5].

The Jefferson Scale of Empathy (JSE) was originally designed for medical students and entitled the Jefferson Scale of Physician Empathy (JSPE), it was subsequently modified to be applicable to not only medical students, but also to the broader populations of practicing physicians and other health professions students and practitioners. Thus, it was renamed as the Jefferson Scale of Empathy (JSE) and there were three versions to be used by physicians, other health professionals and students. The HP-Version used for physicians and other health professionals, while the S-version used for medical students and HPS-version was modified for students in all health professions other than medicine [9]. The content in the three versions was very similar with only minor modifications to make the items appropriate for the target groups. There were several studies has been validated and reported the empathy status using the physician version (JSE-HP) [10], medical students version (JSE-S) [11] and healthcare professional student version (JSE-HPS) [12]. The studies of the empathy level among dental students in different countries reported that a statistically significant difference was found between the study years of the student in most studies [6, 12–14]. Regarding the empathy scores across the gender, the female had higher empathy scores than males in studies [13, 15] while other studies reported higher scores in males [11, 14]. Previous studies also suggested that the experiences of the students in the clinical training might be associated with the declination of their empathy level [16]. In addition, the differences in cultures and education systems among the countries may influence empathy scores and outcomes [8]. Only JSE-S version in Thailand reported that 57% of medical students were below-average levels of empathy, and students’ mental health, training experiences and depersonalization influenced their empathy level [16, 17]. Low empathy level might reduce the satisfactory in patient-physician relationship which can affect the successful treatment [18]. Although The JSE-S version has been validated among Thai medical students [19], the validity and reliability of the JSE-HPS version have not been determined for Thai dental students.

Therefore, this study aimed to investigate the psychometric properties of the Jefferson Scale of Empathy - Healthcare Professions Student version (JSE-HPS) in a sample of Thai dental students and compare the empathy scores across sex, universities, and study year, which would benefit future dental curriculum development to establish empathy in dental students.

## Methods

This cross-sectional study has been approved by the Human Research Ethics Committee of the Faculty of Dentistry, Chulalongkorn University (HREC-DCU 2021-071), and informed consent was acquired from the participants in the study. The participants’ responses were kept confidential and were not linked to their identities. The data analysis was performed in block form, rather than individually, to assure anonymity and confidentiality. The recruitment procedure was started in December 2021 and ended in March 2022.

### Population and sample

The minimal sample size calculation was based on a previous study [12], which reported a standard deviation of 8.59. To estimate the finite population means by n4studies [20], the required sample size was calculated to be 411 dental students. This study was conducted on 1st to 6th-year undergraduate dental students enrolled in six dental universities of the academic year 2021. The inclusion criteria comprised of students from both gender, must be 18 years old and above, and must have completely filled up the questionnaire. The students who declined to participate or provided incomplete information were excluded. The consecutive sampling technique was used to recruit the participants who were available to participate in the study until the required sample size was achieved. Compensating for an estimated 5% of incomplete information, the total number of subjects comprises 432 dental students.

### Research instruments

An online questionnaire which consists of two parts was used to collect the data. The first part was demographic characteristics including gender, age, university, and year of study. The other part was the JSE-HPS questionnaire. The JSE-HPS (Thai version) was developed under the permission of the original developer [9]. The JSE-HPS English version was translated according to the standard guideline for the cross-cultural adaptation process. It contained 20 items with a 7-point Likert scale (strongly disagree = 1, strongly agree = 7). For the JSE-HPS score, the ten positively worded statements were directly scored (the higher score, the higher empathy) while the ten negative statements were reverse scored (the lower score, the higher empathy) (Additional file 1). Therefore, the sum scores ranged from 20 to 140 and a high score indicates greater empathy in caring for the patient. The draft Thai version of JSE-HPS was pilot-tested on 5 dental students to examine the comprehensibility of the questionnaire. Based on the feedback, the final Thai version of JSE-HPS was revised by the expert who approved the questionnaire. A second answer to the questionnaire was performed on 20 dental students 1–2 weeks after the first answer for the test-retest reliability.

### Data collection

The online questionnaires were sent to the representative of dental students in five public dental schools: Chulalongkorn University (CU), Chiang Mai University (CMU), Khon Kaen University (KKU), Prince of Songkla University (PSU), Mahidol University (MU), and one private dental school was Rangsit University (RSU). The undergraduate dental students were asked about their demographic characteristics and 20 items of the JSE-HPS scale.

### Statistics analysis

The data analysis was performed by using the SPSS version 22.00 software (IBM Corp). The mean (standard deviation) of empathy scores and percentage of gender, age, university, and year of study were analyzed with descriptive statistics. Independent samples t-test and one-way ANOVA were used in the comparison of the mean JSE-HPS scores among group differences. Principal Component Analysis (PCA) was performed to explore the underlying factors of JSE-HPS in dental students. The Kaiser-Meyer-Olkin test (KMO) was conducted in measuring the sampling adequacy of greater than 0.7. An eigenvalue of greater than 1 was used to determine retaining factors in PCA. The reliability of the Thai version of JSE-HPS was investigated by analyzing its internal consistency and reliability (test-retest). The internal consistency was examined by Cronbach’s alpha, as well as the corrected item-total correlation coefficients. The intra-correlation coefficient (ICC) was assessed to measure the agreement level between the responses to the first and second questionnaires.

## Results

Four hundred and thirty-nine dental students were recruited of which 72.9% of the participants were female. Out of them, 92.7% (407) studied at public universities which were Chulalongkorn University (CU) (18.7%), Mahidol University (MU) (31.2%), Khon Kaen University (KKU) (16.4%), Chiang Mai University (CMU) (14.6%), and Prince of Songkla University (PSU) (11.8%) while 7.3% [32] were from private university: Rangsit University (RSU). The mean age of the students in this study was 21.75 ± 2.11. The highest participation rate was in the fourth year (21.9%) and the lowest was in the third year (10.2%). The overall internal consistency value was 0.83 and ICC was 0.82 for test-retest reliability analysis. The descriptive statistics of the study were summarized in Table 1.

**Table 1:** Descriptive statistics of the JSE-HPS among dental students (n = 439)

Parameters of sum scores	Statistics
Mean	114.30
Standard deviation (SD)	13.07
Median of score	116.00
Variance	170.71
Cronbach’s alpha coefficient	0.83
Test-retest reliability (ICC)	0.82

We performed the principal component analysis (PCA) with the varimax rotation method to investigate the correlations between the variables or factors of the JSE-HPS version in this study. The overall index (0.87) of the Kaiser-Meyer-Olkin analysis indicated an adequate number of participants for this analysis, and Bartlett’s sphericity test showed a correlation among the factors (X^2^ = 2268.62, *p* < 0.001). Among the five factors of the eigenvalues > 1, only the first three factors met the criteria in which at least three items per factor are necessary for a stable factor [21]. According to this principle, the remaining two factors might not be stable as the first three factors. Therefore, three factors were extracted by PCA, and these three factors explained 42.6% of the total variance in this study.

The largest proportion (27.4%) of the variance was explained by factor 1 (Compassionate care) which included eight items with factor loading values greater than 0.35 except item 19. Factor two (Perspective Taking) comprised ten items with factor loadings greater than 0.35 which accounted for 8.2% of the total variance. Factor three (Standing in the patient’s shoes) which consists of the two items with factor loadings ≥ 0.35 attributed the 7% of the variance. The Cronbach’s alpha values for these three components were 0.76, 0.77, and 0.64 in the internal consistency analysis (Table 2).

**Table 2:** Factor coefficient, mean (SD) for PCA with corrected item-total correlation and Cronbach’s alpha values JSE-HPS (n = 439)

Items	Factor coefficient			Mean score (SD)	*r*_***i−t***_
	1	2	3		
Attentiveness to patients’ personal experiences does not influence treatment outcomes. (-) Q8	**0.752**	0.114	-0.078	1.96 (1.331)	0.596
Healthcare providers’ understanding of their patients’ feelings and the feelings of their patients’ families do not influence treatment outcomes. (-) Q1	**0.705**	-0.054	-0.050	2.13 (1.705)	0.501
Patients’ illnesses can be cured only by targeted treatment; therefore, healthcare providers’ emotional ties with their patients do not have a significant influence in treatment outcomes. (-) Q11	**0.671**	0.210	0.114	2.03 (1.315)	0.573
Attention to patients’ emotions is not important in-patient interview. (-) Q7	**0.643**	0.173	0.086	1.50 (1.016)	0.529
Asking patients about what is happening in their personal lives is not helpful in understanding their physical complaints. (-) Q12	**0.637**	0.292	0.143	1.81 (1.097)	0.558
I believe that emotion has no place in the treatment of medical illness. (-) Q14	**0.621**	0.272	-0.038	1.74 (1.135)	0.518
Healthcare providers should not allow themselves to be influenced by strong personal bonds between their patients and their family members. (-) Q18	**0.376**	0.063	0.160	3.73 (1.663)	0.291
I do not enjoy reading non-medical literature or the arts. (-) Q19	**0.326**	0.149	0.174	1.72 (1.380)	0.267
Empathy is a therapeutic skill without which a healthcare provider’s success is limited. (+) Q15	0.159	**0.662**	0.024	5.84 (1.250)	0.528
I believe that empathy is an important factor in patients’ treatment. (+) Q20	0.360	**0.621**	0.107	6.26 (0.990)	0.581
Healthcare providers should try to think like their patients in order to render better care. (+) Q17	-0.024	**0.596**	0.005	5.12 (1.678)	0.394
Healthcare providers should try to stand in their patients’ shoes when providing care to them. (+) Q9	0.002	**0.579**	0.093	5.61 (1.522)	0.410
Healthcare providers’ understanding of the emotional status of their patients, as well as that of their families is one important component of the healthcare provider-patient relationship. (+) Q16	0.468	**0.514**	-0.015	6.13 (1.032)	0.514
Patients value a healthcare provider’s understanding of their feelings which is therapeutic in its own right. (+) Q10	0.356	**0.514**	0.130	5.94 (1.063)	0.508
Understanding body language is as important as verbal communication in healthcare provider–patient relationships. (+) Q4	0.074	**0.509**	0.114	5.83 (1.306)	0.368
Patients feel better when their healthcare providers understand their feelings. (+) Q2	0.363	**0.488**	-0.085	6.56 (0.750)	0.461
A health care provider’s sense of humour contributes to a better clinical outcome. (+) Q5	0.181	**0.475**	-0.005	5.60 (1.316)	0.363
Healthcare providers should try to understand what is going on in their patients’ minds by paying attention to their non-verbal cues and body language. (+) Q13	0.175	**0.449**	0.104	5.80 (1.385)	0.372
Because people are different, it is difficult to see things from patients’ perspectives. (-) Q6	0.057	0.001	**0.866**	4.60 (1.843)	0.483
It is difficult for a healthcare provider to view things from patients’ perspectives. (-) Q3	0.119	0.204	**0.796**	2.97 (1.529)	0.483
Eigenvalue	5.4	1.6	1.4		
Cronbach’s alpha coefficient	0.76	0.77	0.64		
Variance (%)	27.4	8.2	7		

Factor 1 (Compassionate care); Factor 2 (Perspective Taking); Factor 3 (Standing in Patient’s Shoes); ***r***_***i−t***_ = corrected item total correlation

The total actual scores ranged from 20 to 140, with a mean empathy score of 114.30 ± 13.06. The mean scores for each item of the positive questions ranged from 5.12 to 6.56 and ranged from 1.5 to 4.6 for negative questions (Table 3). The highest mean value was 6.56 in a positive questionnaire (Q 2) and the lowest was 1.50 in a negative item (Q 7). Although females had a slightly higher mean score (115.1 ± 11.53) compared to males (112.8 ± 15.38), a statistically significant difference was not found (*p* = 0.085). The mean score of the sixth-year students was the highest (116.7 ± 11.20), followed by second-year students (116.3 ± 9.82). The mean difference score across the study program, phase of training, grade, university, region, type of university, and study year was not statistically significant (Table 3).

**Table 3:** Mean empathy scores of the JSE-HPS in group differences

Factors	N	%	Mean score (SD)	p-value
Gender				
Male Female	119 320	27.1 72.9	112.8 (15.38) 115.1 (11.53)	0.085^a^
Study program				
Regular International	403 36	91.8 8.2	114.4 (12.83) 115.4 (11.42)	0.670^a^
Phase of training				
Pre-clinical Clinical	173 266	39.4 60.6	114.7 (13.05) 114.3 (12.51)	0.762^a^
Grade				
2.00-2.49 2.50–2.99 3.00-3.49 3.50-4.00	12 49 180 198	2.7 11.2 41.0 45.1	120.2 (14.21) 116.8 (8.70) 113.1(13.38) 114.9 (12.71)	0.087^b^
University				
CU MU KKU CMU PSU RSU	82 137 72 64 52 32	18.7 31.2 16.4 14.6 11.8 7.3	113.0 (15.00) 114.0 (11.25) 116.6 (14.63) 115.9 (11.71) 113.5 (10.82) 114.3 (12.54)	0.521^b^
Region of University				
Central Northeast North South	251 72 64 52	57.2 16.4 14.6 11.8	113.7 (12.71) 116.6 (14.63) 115.9 (11.71) 113.5 (10.82)	0.281^b^
Type of University				
Public Private	407 32	92.7 7.3	114.5 (12.74) 114.3 (12.54)	0.945^a^
Year of Study				
First Second Third Forth Fifth Sixth	61 67 45 96 80 90	13.9 15.3 10.2 21.9 18.2 20.5	114.9 (16.00) 116.3 (9.82) 112.4 (12.95) 113.1 (14.70) 113.0 (10.53) 116.7 (11.20)	0.177^b^

^a^ Independent samples t-test (*p* < 0.05 statistically significant)

^b^ One-way analysis of variance (ANOVA) (*p* < 0.05 statistically significant)

## Discussions

The finding of the present study demonstrated the satisfactory reliability and validity of the JSE-HPS (Thai version) among Thai dental students. The Cronbach’s alpha value (0.83) demonstrated the acceptable internal consistency of the JSE-HPS (Thai version) similar to that of other studies [6, 8, 13]. The factorial analysis of this study confirmed the construct validity of the JSE-HPS (Thai version) among Thai dental students based on the findings of the original version [22] and the Thai medical student version [19].

In our study, there were three main domains of empathy including factor 1(Compassionate care), factor 2 (Perspective taking), and factor 3 (Standing in the patient’s shoes). Among those, compassionate care was the first factor to emerge in the PCA analysis demonstrating the major dimension of empathy. This finding is in accordance with studies in Brazil [1], Iran [23], Nigeria [6]. However, it was in contrast with the Korean, Japanese, Malaysian, and original versions [4, 8, 22, 24] in which Perspective Taking was the first dimension. The explanation for this finding may be related to the cultural differences among the countries. The presentation of most Thai interactions is honest and respectful, and the Thai are bound for sincere and deep reciprocal relationships. Reciprocity of kindness, particularly the value of being sympathetic is a highly valued character trait in Thai society. The influence of religion and Buddhism concepts of compassion has also been pointed out. It is supposed that these culture-specific features can lead to differences in empathy scores among different populations.

The perspective-taking component was the second factor in our analysis while the other studies indicated factor one [4, 8] and factor three [1, 6]. The results may be influenced by both cultural differences and the school curriculum related to bioethics, patient safety, and the interdisciplinary field of humanities [1]. “Standing in patient’s shoes” is the third factor obtained from factor analysis which is consistent with the study of Thai medical students and other studies [4, 23, 24].

The mean empathy scores among Thai dental students corroborate other healthcare professional students such as nurses [25, 26], medical [19, 24, 27], and pharmacy [28] using the same scale. Compared with dental students, the mean score of Thai dental students was higher than the scores among dental students in Malaysia [4], India [11, 12, 15], Saudi Arabia [29, 30], Nepal [31], and Nigeria [6]. However, it had a lower score compared with dental students in the USA [32]. This finding might be related to different cultures, curriculums, training experiences and study environments. In this study, the female had higher mean scores compared to the male which is similar to other studies [6, 9, 15, 19, 24–26, 28–30, 32, 33]. In contrast, some studies reported that male students had much more empathy scores than female students such as dental students in India [11, 14, 34], Malaysia [4], and Nepal [31]. The gender difference has been accounted to biology, interpersonal style in caring, socialization, or cultural expectations about gender roles.

Regarding the study year of the student, although there were no significant differences in empathy scores among different study years, the mean empathy score for the sixth year was found to be the highest when compared to other years of study. However, the study conducted in Southern Thailand reported that the total empathy score of the fourth-year students was highest compared to the fifth and sixth year students [16]. In addition, this study showed that there was no statistically significant different between the preclinical and clinical year, whereas the preclinical years had significantly higher score than the clinical years in previous study among Thai medical students [17]. It might be due to having different academic stress levels, opportunities to take part in patient care, cogitating the dentist-patient relationship and using different study instruments.

Among the universities, the mean empathy scores of Khon Kaen university located in rural areas were higher than the other universities though this finding was not statistically significant. This finding may be a result of the different cultural norms in Northeast Thailand which had a high empathic engagement during doctor-patient relationships than in other areas [34]. Moreover, there was no significant difference in the empathy scores of the students between the public and private universities. The possible explanation might be that the Thai dental school curriculum mostly focuses on professional practice, competency, and comprehensive patient-centred care at present [35]. Public dental schools as well as private schools can facilitate clinical practice skills, communication skills, and cultural competence in community outreach experience from the early years of study.

This study had certain limitations. The participants were recruited by consecutive sampling; thus, sampling bias needs to be considered. Nevertheless, our samples were distributed in the different areas of dental schools. Due to a cross-sectional design, therefore, the mental health of the student, their stress level and the high participation rate of the female respondents might influence the findings of this study. Other issues regarding the use of online questionnaires such as misunderstanding of the questions and limited information about the characteristics of the non-respondents needed to be considered. Therefore, we cautiously make a generalization from the study sample to the experiences of the student population. A longitudinal study design with a wide-range representative sample or qualitative study are recommended for investigating the associated factors and examining changes in empathy during dental education.

## Conclusions

The findings demonstrated that the JSE-HPS Thai version was a sound instrument in psychometric properties to measure the empathy level of dental students. The mean JSE-HPS score among Thai dental students is higher than those reported in other dental studies. Integrating empathic elements into the dental curriculum will help student learning to be more effective and improve treatment outcomes.

## Electronic supplementary material

Below is the link to the electronic supplementary material.

Supplementary Material 1: Questionnaire

## Data Availability

The datasets generated and/or analyzed during the current study are available from the corresponding author on reasonable request.
