# Supplementary material for: Empathy level towards patients among thai dental students: a cross-sectional study
Source: BMC Oral Health. 2023 Mar 30;23:184. doi: 10.1186/s12903-023-02891-6 (PMC10061995; doi:10.1186/s12903-023-02891-6)
Supplement: Supplementary file 1 — Supplementary Material 1: Questionnaire [file 12903_2023_2891_MOESM1_ESM.pdf]

Jefferson
Thomas Jefferson University
HOME OF SIDNEY KIMMEL MEDICAL COLLEGE

Jefferson Scale of Empathy

Health Professions Student version (HPS- version)

Use a ball-point pen. Mark one response for each item below.
For *ID Code*, write numerals completely inside the boxes, one numeral to a box.

Name (optional) ____________________________________ **ID Code**............ ☐☐☐☐☐☐☐☐☐

Date ____/____/________

Age:

☐ < 19 ☐ 19 - 21 ☐ 22- 24 ☐ 25 - 27 ☐ 28 - 30 ☐ 31 - 33 ☐ 34 - 36
☐ 37 - 39 ☐ 40 - 42 ☐ 43 - 45 ☐ 46 - 48 ☐ 49 - 51 ☐ >51

Gender:

☐ Male ☐ Female

What is your degree program?

☐ Bioscience/Medical technology ☐ Counseling/Psychology ☐ Dentistry
☐ Diagnostic Imaging ☐ Nursing ☐ Nurse Practitioner
☐ Occupational Therapy ☐ Ophthalmology/Optometry ☐ Pharmacy
☐ Physical Therapy ☐ Physician Assistant ☐ Public Health
☐ Other ____________________

Year in this program:

☐ 1st year ☐ 2nd year ☐ 3rd year ☐ 4th year ☐ > 4th year

Please leave *Optional* fields blank unless otherwise instructed.

Optional field #1 .... ☐☐☐☐

Optional field #2 .... ☐☐☐☐

PLEASE CONTINUE ⇨⇨⇨

S E L F - S C O R E

© Thomas Jefferson University, 2009 All rights reserved 140716 (ver. 1.2.1)

Jefferson Scale of Empathy

Health Professions Student version (HPS-version)

Instructions: Using a ball-point pen, please indicate the extent of your agreement or disagreement with *each* of the following statements by marking the appropriate circle to the right of each statement.

Please use the following 7-point scale (*a higher number on the scale indicates more agreement):*
Mark <u>one and only one</u> response for each statement.

1-------2-------3-------4-------5-------6-------7
Strongly Disagree ***Strongly Agree***

	1	2	3	4	5	6	7
1. Health care providers' understanding of their patients' feelings and the feelings of their patients' families does not influence treatment outcomes.	○	○	○	○	○	○	○
2. Patients feel better when their health care providers understand their feelings.	○	○	○	○	○	○	○
3. It is difficult for a health care provider to view things from patients' perspectives.	○	○	○	○	○	○	○
4. Understanding body language is as important as verbal communication in health care provider - patient relationships.	○	○	○	○	○	○	○
5. A health care provider's sense of humor contributes to a better clinical outcome.	○	○	○	○	○	○	○
6. Because people are different, it is difficult to see things from patients' perspectives.	○	○	○	○	○	○	○
7. Attention to patients' emotions is not important in patient interview.	○	○	○	○	○	○	○
8. Attentiveness to patients' personal experiences does not influence treatment outcomes.	○	○	○	○	○	○	○
9. Health care providers should try to stand in their patients' shoes when providing care to them.	○	○	○	○	○	○	○
10. Patients value a health care provider's understanding of their feelings which is therapeutic in its own right.	○	○	○	○	○	○	○
11. Patients' illnesses can be cured only by targeted treatment; therefore, health care providers' emotional ties with their patients do not have a significant influence in treatment outcomes.	○	○	○	○	○	○	○
12. Asking patients about what is happening in their personal lives is not helpful in understanding their physical complaints.	○	○	○	○	○	○	○
13. Health care providers should try to understand what is going on in their patients' minds by paying attention to their non-verbal cues and body language.	○	○	○	○	○	○	○
14. I believe that emotion has no place in the treatment of medical illness.	○	○	○	○	○	○	○
15. Empathy is a therapeutic skill without which a health care provider's success is limited.	○	○	○	○	○	○	○
16. Health care providers' understanding of the emotional status of their patients, as well as that of their families is one important component of the health care provider - patient relationship.	○	○	○	○	○	○	○
17. Health care providers should try to think like their patients in order to render better care.	○	○	○	○	○	○	○
18. Health care providers should not allow themselves to be influenced by strong personal bonds between their patients and their family members.	○	○	○	○	○	○	○
19. I do not enjoy reading non-medical literature or the arts.	○	○	○	○	○	○	○
20. I believe that empathy is an important factor in patients' treatment.	○	○	○	○	○	○	○

THANK YOU!

© Thomas Jefferson University, 2009 All rights reserved 140716 (ver. 1.2.1)
